# Interactive tool for clustering and forecasting patterns of Taiwan COVID-19 spread

**DOI:** 10.1371/journal.pone.0265477

**Published:** 2022-06-30

**Authors:** Mahsa Ashouri, Frederick Kin Hing Phoa

**Affiliations:** Institute of Statistical Science, Academia Sinica, Taipei, Taiwan; Southern Illinois University, UNITED STATES

## Abstract

The COVID-19 data analysis is essential for policymakers to analyze the outbreak and manage the containment. Many approaches based on traditional time series clustering and forecasting methods, such as hierarchical clustering and exponential smoothing, have been proposed to cluster and forecast the COVID-19 data. However, most of these methods do not scale up with the high volume of cases. Moreover, the interactive nature of the application demands further critically complex yet compelling clustering and forecasting techniques. In this paper, we propose a web-based interactive tool to cluster and forecast the available data of Taiwan COVID-19 confirmed infection cases. We apply the Model-based (MOB) tree and domain-relevant attributes to cluster the dataset and display forecasting results using the Ordinary Least Square (OLS) method. In this OLS model, we apply a model produced by the MOB tree to forecast all series in each cluster. Our user-friendly parametric forecasting method is computationally cheap. A web app based on R’s Shiny App makes it easier for practitioners to find clustering and forecasting results while choosing different parameters such as domain-relevant attributes. These results could help in determining the spread pattern and be utilized by medical researchers.

## Introduction

The Coronavirus Disease 2019 (COVID-19) from Wuhan (Hubei, China), which started spreading quickly in late December 2019, was announced as an outbreak by the public health emergency of international in January 2020 and a pandemic by the World Health Organization (WHO) on March 11, 2020. It transmits from person to person and causes symptoms like high fever, cough, and shortness of breath after a 2-to-14-day infection period [1]. On December 15, 2020, more than 72.8 million people were confirmed by COVID-19, with 742 cases confirmed in Taiwan. Confirmed cases grew exponentially across all continents [2]. The world has changed dramatically ever since the first case broke out, and many countries have encountered multiple crises, such as health crises, financial crises, and economic collapses [3]. At that time, Taiwan had successfully curbed the spread for more than a year since the outbreak started.

Taiwan center of disease control reported the first confirmed infection case on January 21, 2020, a 50-year-old woman who was a teacher in Wuhan. Due to early responses and active contact tracing policies, Taiwan managed to contain the spread successfully with a record of 250 consecutive days without any locally transmitted cases. However, Taiwan started to face a sharp surge of confirmed cases in late April 2021 [4]. The policy-making and spread patterns of the disease depend on many factors (such as environmental factors [5]), which may not follow the previously available models. Therefore, creating a more efficient and accurate interactive analytical tool is essential in identifying the spread pattern and providing helpful information to enact effective policies.

Time series clustering is essential to determine similarities and/or differences in the behavior of COVID-19 across cities, states, or countries, and it is advantageous in selecting forecasting models. [6] measured the similarity of the COVID-19 time series between states using the dynamic time warping distance (DTW) as the similarity matrix and applied a hierarchical clustering approach to analyze the behavioral relationships in the United States (US) pandemic. As a result, they found different pandemic behaviors in eastern and western zones. [7] suggested a non-negative matrix factorization (NMF) followed by a k-means clustering procedure on the coefficients of the NMF basis to cluster the US states into different communities. Their method not only has the advantage of capturing patterns, but it has also reflected the spread and control of the pandemic by July 25, 2020.

[8] used an unsupervised machine learning technique to identify COVID-19 cases. They applied a lung radiography dataset to the Robust Continuous Clustering algorithm (RCC) to identify confirmed patients.

Forecasting the pattern of the COVID-19 pandemic is critical to health services, health policymakers, healthcare providers, and epidemiologists. Various time series approaches aim to forecast the COVID-19 pandemic using statistical modeling. For example, [9] proposed a time series statistical approach to predict the short-term behavior of COVID-19. They applied multiplicative trend to forecast the number of confirmed cases and deaths globally and presented a 10-day-ahead competitive forecast over four months. [10] introduced an objective approach to predict the continuation of COVID-19. They produced forecasts using models from the exponential smoothing family suitable for the short-term time series. [2] presented a simple interactive non-linear method to forecast the number of confirmed cases. Their method took the expected recoveries and deaths into account to determine the maximum daily growth rate. Finally, [11] suggested a simplified and accurate method using fast linear regressions with only a few parameters to forecast deaths, which can consider the effect of many complexities of the epidemic process.

### R’s Shiny app

R’s Shiny app [12] is a package from RStudio [13] developed for an easier and more efficient result visualization. This web-based application allows users to change the model parameters and interact with results. In the COVID-19 subject, many researchers published medical and epidemiological research regarding interactive data analysis and visualization with the R Shiny framework.

For instance, the COVID-19 tracker [14] in R’s Shiny package provides more context for daily headlines and a fresh perspective of historical turning points.

[15] developed a COVID-19 worldwide web-based application using R’s Shiny package. They design the tool for the country-specific analysis to visualize epidemiological pandemic indicators. [16] suggested a COVID-19 watcher of the updated information for medical and public use. Their tool aggregated the data from different resources and visualized them using an online dashboard.

This research proposes an interactive web-based R’s Shiny app to cluster and forecast Taiwan COVID-19 time series while benefiting from domain-relevant attributes. Our tool helps users choose from various parameters to interact with results. For example, users can identify possible domain-relevant splitting variables of interest.

The dataset contains the number of confirmed cases in the cities, townships, and districts of Taiwan. The data was collected from Taiwan Centers for Disease Control (CDC)and contains 183 daily series with a length of 155 from January 1 to June 4, 2021. We assume that this governmental data is legitimate and trustworthy.

Our app can also be used to analyze COVID-19 time series data of any places in the rest of the world, only if the dataset follows the same structure below. There should be eight columns in the dataset corresponding to administrative types, city name, a YES/NO on whether the city has an airport, a YES/NO on whether the case is imported or local otherwise, the number of cases, the region category, the number of population, and the date. Among them, all except the number of cases, the number of population, and the dates are categorical entries. In addition, all rows are arranged in the ascending order of the dates for each city. Finally, the first row should be the name of the titles of these eight columns.

## Methodology

To cluster and forecast COVID-19 time series, we applied the method suggested by [17], and we will briefly explain it in this section. This clustering approach applies domain-relevant attributes and time series temporal patterns (trend, seasonality, and autocorrelation). Domain-relevant attributes are cross-sectional attributes that link time series into sub-groups. For example, the sales volume of items in a supermarket can be divided into different sub-groups. Similarly, the COVID-19 cases can also be grouped based on geographical features.

This method based on the model-based partitioning tree (MOB) [18] is automated for clustering large collections of time series. It consists of fitting local parametric models into different subsets based on a recursive partitioning algorithm. The parameters and split points are estimated using an objective function and a greedy forward search. To determine which variable should be used for partitioning, we test each model score for parameter instability in each node. Each node of the resulting tree is associated with a parametric statistical model. When using the MOB algorithm, we need to specify the outcome, the predictors, the splitting variables, and the ‘fit’ function. The next part will discuss how the ordinary least squares (OLS) model is used as the ‘fit’ function within the MOB framework.

To capture time series temporal patterns, [17] suggested an OLS model with predictors to model their trend, seasonality, and autocorrelation. This model is parametric and flexible in trend shapes (e.g., linear, quadratic) and seasonal patterns (e.g., seasonal dummies or a smooth function for slowly changing seasonality). These predictors allow incorporating external attributes valuable for clustering or forecasting time series. For instance, we can include the ‘Easter’ dummy variable indicating the timing of Easter.

$$\begin{array}{c} Y=Trend+Season+AR(p)+External\;data+error, \end{array}$$ (1)

Where *AR*(*p*) is a weighted average of lags in order *p*, and *p* can be equal to seasonality order or specified based on the data type and domain knowledge. As an example, Eq 1 can be written as:

$$\begin{array}{c} \begin{array}{cc} y_{t} & =\alpha_{0}+\alpha_{1}f\left(t\right)\\ & +\beta_{1}Season_{1t}+\beta_{2}Season_{2t}+\cdots +\beta_{m-1}Season_{(m-1)t}\\ & +\gamma_{1}y_{t-1}+\gamma_{2}y_{t-2}+\cdots +\gamma_{p}y_{t-p}\\ & +\delta z_{t}+\epsilon_{t}, \end{array} \end{array}$$ (2)

where *y*_*t*_ (*t* = 1, 2, …, *T*) is the value of series at time *t*, *f*(*t*) is a function of the time index that captures trend (e.g., linear, quadratic), *Season*_*jt*_ is a dummy variable taking value 1 if time *t* is in season *j*, *m* is the number of seasons (e.g., for a daily time series with day-of-week seasonality, *m* = 7), and *z*_*t*_ is the external data at time *t*. Furthermore, *y*_*t*−*j*_ is the *j*th lagged value. One advantage of OLS models is the interpretability of coefficients. The contribution of each feature to the output will be equal to its coefficient. For example, if there is a linear trend, *α*_1_ measures the changes in *y*_*t*_ from one period to the next due to the passage of time while holding other variables in the model constant. As another example, with quadratic trend, *α*_1_ *f*(*t*) would be $\alpha_{1}^{\prime }t+\alpha_{1}^{\prime \prime }t^{2}$ means when $\alpha_{1}^{\prime }$ and $\alpha_{1}^{\prime \prime }$ are positive, the trend is increasing while holding other variables in the model constant.

Using the MOB partitioning tree and pseudo-R notations with partitioning variables [*Z*_1_, …, *Z*_*q*_], Eq 2 can be written as:

$$\begin{array}{c} \begin{array}{cc} y_{t} & =\alpha_{0}+\alpha_{1}f\left(t\right)\\ & +\beta_{1}Season_{1t}+\beta_{2}Season_{2t}+\cdots +\beta_{m-1}Season_{(m-1)t}\\ & +\gamma_{1}y_{t-1}+\gamma_{2}y_{t-2}+\cdots +\gamma_{p}y_{t-p}\\ & +\delta z_{t}|Z_{1}+\cdots +Z_{q}. \end{array} \end{array}$$ (3)

This approach creates clusters with the same domain-relevant attribute profile and the similar trend, seasonality, and autocorrelation pattern. Based on this approach, we can cluster the time series using Algorithm 1.

**Algorithm 1:** MOB time series clustering algorithm

- **Zero time series:** separate ‘all zero’ time series
- **Normalize the series:** subtract the mean and divide the standard deviation
- **MOB tree:** run the tree on the series using Eq 3
- **Prune the MOB tree:** stop the tree when reaching the best improvement on Mean Square Error (MSE), tree simplicity, and AIC [19] or BIC [20]
- **Coefficient plot:** compare OLS models in non-neighboring clusters and check their differences/similarities

Finally, we computed forecasts by one linear model in each cluster produced by the MOB partitioning tree. We apply the same linear model for series in the same cluster to produce forecasts. We generate forecasts at fixed time *t* with *h* steps ahead (the lagged values of *y* are replaced by their forecasted values if they occur in periods after the forecast origin). We also compare our OLS forecast results with the Exponential Smoothing (ETS) approach. For running ETS, we applied functions ‘est’ forecast package [21] in R. We run this function independently on each series. Then, we use the average of Root Mean Square Errors (RMSEs) and Mean Absolute Error (MAE) across all series and display box and density plots for forecast errors. We define the forecast error as the difference between the observed value and its forecast. For better visualization, we do not plot the outliers.

## Clustering and forecasting Taiwan COVID-19 confirmed cases

The collected dataset includes 183 daily series (cities, townships, and districts) with a length of 161 from January 1 to June 10, 2021, and the number excluding zero time series is one. Before running the clustering method, we scaled the data by subtracting the mean and dividing the standard deviation. Additionally, we partition the data into training and test sets, with the last 7 days as our test set and the rest as the training set. Then we combine the training and test sets and update the model and forecast one-week-ahead of the confirmed cases. Note that we update the model in each cluster while keeping the clustering results unchanged.

For Taiwan COVID-19 daily dataset, we included the following predictors in the MOB-based clustering and forecasting OLS model (‘fit’ function): a linear trend, six seasonal dummies, and lags 1 to 7. Also domain-relevant splitting variables includes geographical division, including ‘region’ (6 categories), ‘administrative’ (3 categories), ‘population’ (numeric), ‘imported’ (2 categories), and ‘airport’ (2 categories) (Table 1).

**Table 1:** Domain-relevant attribute categories used in Taiwan COVID-19 confirmed infection cases.

Domain-relevant attributes	Categories
Region	north, east, west, south, null (imported cases)
Administrative	township/city, district, null (imported cases)
Population	numeric—no categories
Imported	yes, no (local cases)
Airport	yes, no (the city has an international airport or not)

### Interactive tool

Table 2 demonstrates the interactive panel inside our tool with three options to choose from, the MOB depth (number of splits +1), prune option, and domain-relevant attributes (splitting variables). Additionally, the ‘choose file to upload’ button lets users upload the desired dataset.

**Table 2:** Taiwan COVID-19 interactive tool panel.

Categories	Application
Choose file to upload	let users upload the Taiwan COVID-19 dataset
MOB depth (number of splits + 1)	changes from ‘no split’ to ‘full tree’, which controls the tree simplicity
Prune option	AIC or BIC
Splitting variables	include all available options for domain-relevant attributes (splitting variables). Options are ‘region’, ‘administrative’, ‘population’, ‘imported’, and ‘airport’
Screenshot	let users screenshot the result

**Fig 1:**
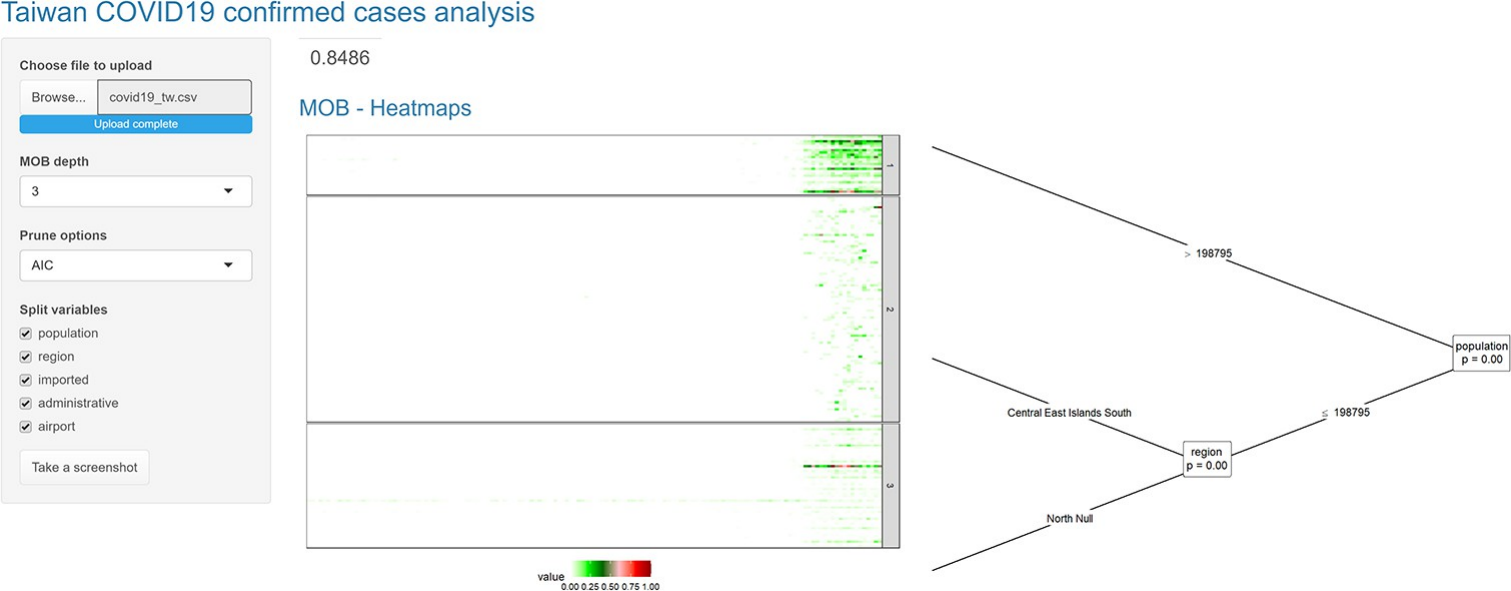
Clustering and forecasting Taiwan COVID-19 confirmed infection cases—Part 1.

**Fig 2:**
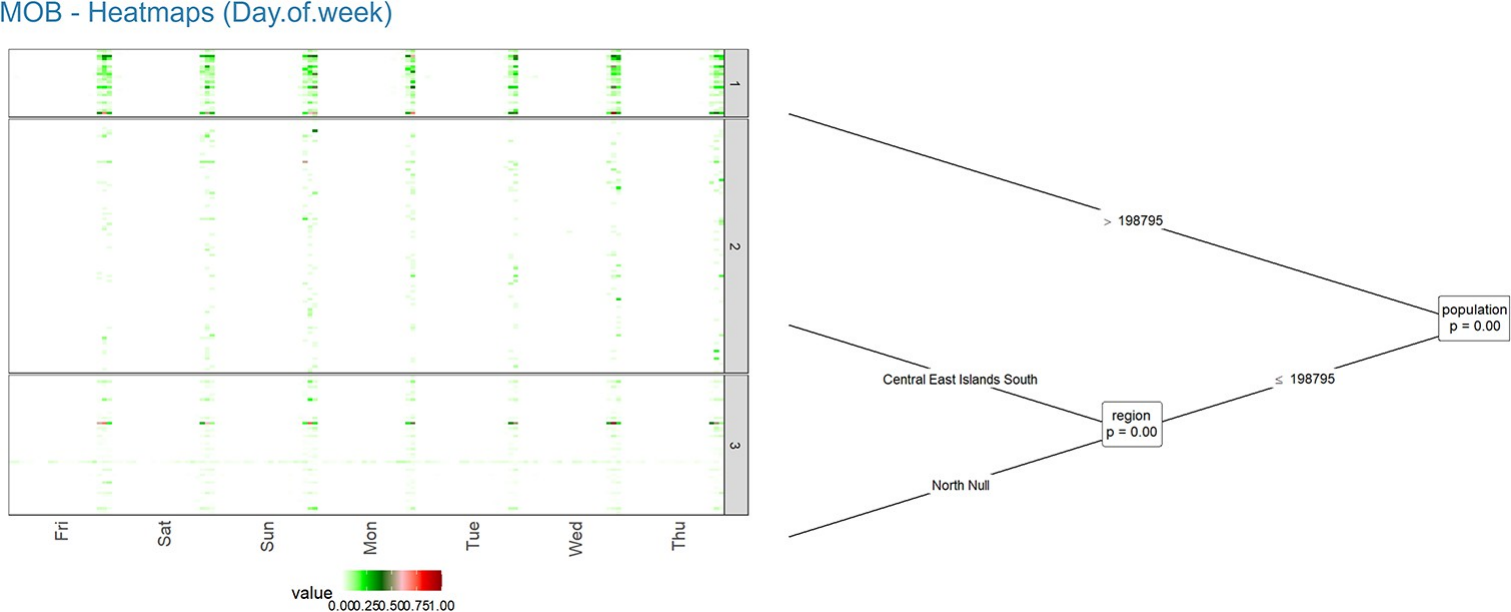
Clustering and forecasting Taiwan COVID-19 confirmed infection cases—Part 2.

**Fig 3:**
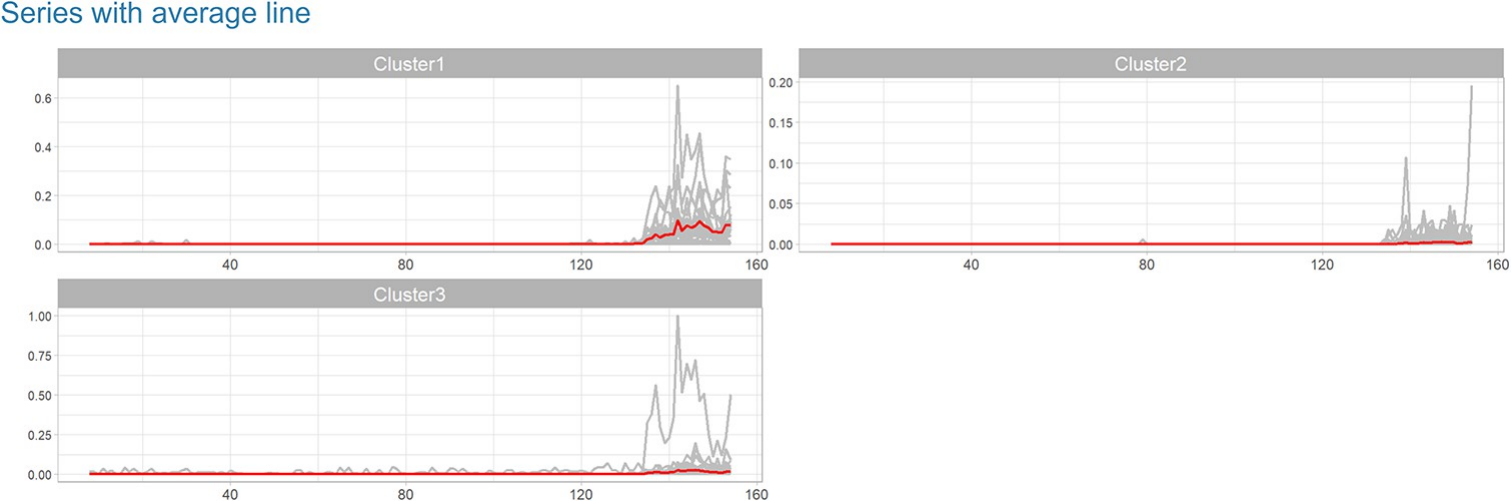
Clustering and forecasting Taiwan COVID-19 confirmed infection cases—Part 3.

**Fig 4:**
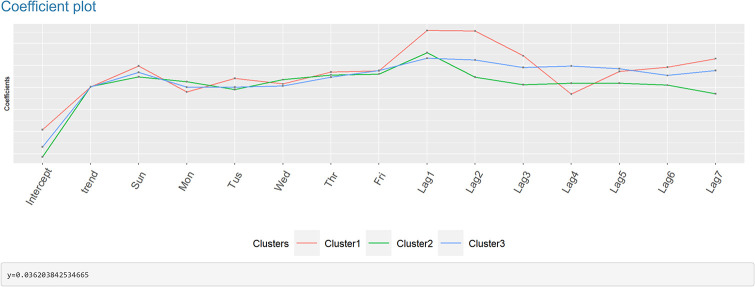
Clustering and forecasting Taiwan COVID-19 confirmed infection cases—Part 4.

**Fig 5:**
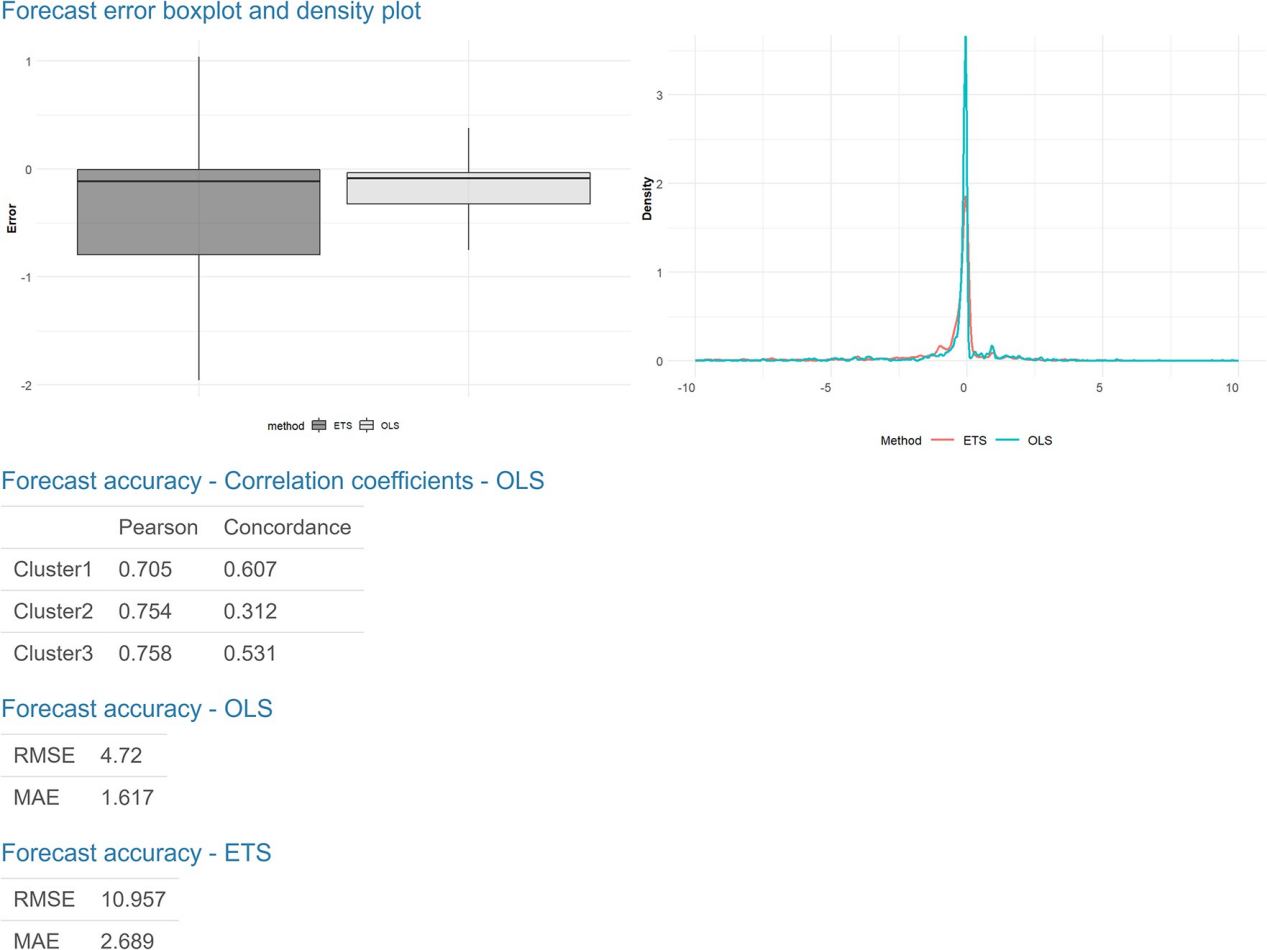
Clustering and forecasting Taiwan COVID-19 confirmed infection cases—Part 5.

**Fig 6:**
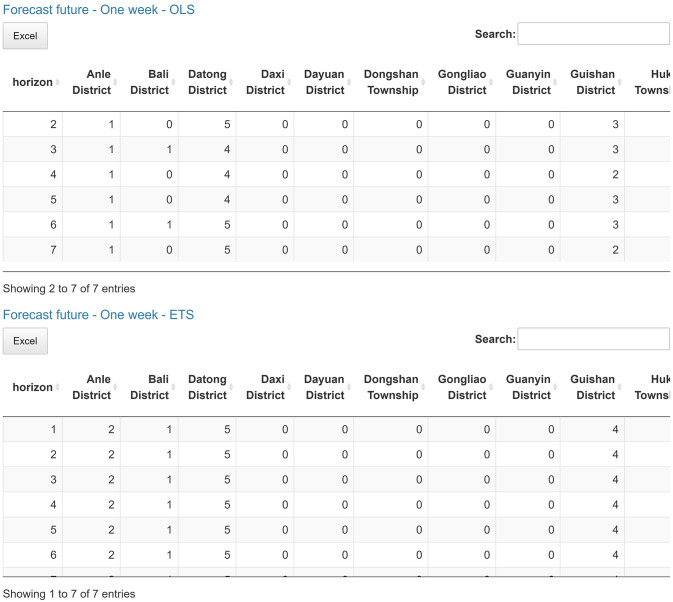
Clustering and forecasting Taiwan COVID-19 confirmed infection cases—Part 6.

Our web-based interactive tool consists of eight parts (displays in Figs 1 to 6). For better visualization, we divide the results into six figures.

The number on the top shows the MSE for all splits in the MOB partitioning tree. The first MOB-heatmap includes two parts (Fig 1). The right part displays the MOB tree, which helps users see domain-relevant attributes and split order accessioned with each cluster based on the specified depth, prune options, and domain-relevant attributes. The left part is the time series heatmap of all clusters, displaying time series patterns. Each row represents one series, and darker color means higher values (color pallet: white, green, and red). Vertical stripes specify similarities among the series in each cluster.

The second MOB-heatmap (Fig 2) is similar to the first plot, except it combines periods into seasonal aggregations to highlight the seasonal effects. In both heatmaps, we order series based on their values. For example, series with higher values in a similar period gather in the same area. Also, based on the number of series in each cluster, the size of the cluster box would be different.

The following plot shows the time series line chart in gray and the average line in red (Fig 3). The coefficient plot displays OLS coefficients for predictors in all clusters (Fig 4). In other words, each line represents one model connecting the coefficients for each predictor. This plot is useful for users to choose the number of clusters. Also, by clicking on the coefficient points, its value will appear in the box below. The final plots, the forecast error box, and density plots, display forecast errors for the OLS and ETS methods on a one-week test set (Fig 5). In the following tables, we first examine the linear models in each cluster and OLS results by computing Pearson [22] and concordance correlation coefficient [23] (between forecasted and observed values). Then we compare OLS and ETS approaches using RMSE and MAE across all series. Lastly, we presented the one-week-ahead forecast results (by updated model on combined training and test sets) of all cities, townships, and districts in Taiwan computed by OLS and ETS models (Fig 6). Users can download the forecasting results in an excel file by clicking on the ‘Excel’ button next to tables.

Figs 1 to 6 demonstrate the screenshots of our interactive tool results of Taiwan COVID-19 confirmed cases.

In Fig 1, in the top left side panel, we chose three as the MOB tree depth (two splits), AIC as the prune option, and all splitting variables as domain-relevant attributes, which resulted in three clusters differing in terms of ‘population’ and ‘region’. Changing options in the panel update results shown in Figs 1 to 6. In Fig 1, the first split divides the series into *population more than 198795* and *population less than 198795*, and for more populated areas, there is no further splits while in the less populated area there is one further split on the ‘region’, shows series in *central, east, south, islands* (up) behave differently from *north, null (imported cases)* (down).

Table 3 represents the final clusters of confirmed cases with the number of series.

**Table 3:** Cluster categories of Taiwan COVID-19 confirmed infection cases by choosing three as the MOB depth, AIC as pruning option, and region, population, imported, administrative, and airport as domain-relevant attributes.

	Cluster categories	Number of series
Cluster 1	Population: more than 198795	26
Cluster 2	Population: less than 198795 Region: central, east, south and islands	103
Cluster 3	Population: less than 198795 Region: north and null (imported cases)	54

The heatmap in this figure shows in early June—when pick started—the number of confirmed cases is higher and more frequent (frequent dark green and red points—‘spiky’ series) in the more populated areas (cluster 1), while the number of confirmed cases is fewer and less frequent (frequent light green points) in the less populated areas (clusters 2 and 3). Also, the diverse distribution of cases (time series temporal patterns), based on populations and regions, is visible between the final clusters.

The heatmap in Fig 2 shows changes in the number of confirmed cases on different days of the week. Based on this plot, the number of reported cases in all clusters is lower on Mondays and Tuesdays and slightly higher on Sundays. Fig 3 shows the line chart of all series with their average (red line) in each cluster. The comparison of the series and the average line in different clusters shows the visible between-cluster variability. Clusters 1 (more populated areas) show more confirmed cases. In cluster 3, the imported case series demonstrates a continuous report of confirmed cases from the beginning of the year. Another series (Wanhua District) shows a high jump in early June when the breakdown started in Taiwan.

Based on the coefficient plot in Fig 4, coefficients in all clusters differ mainly in terms of lags (daily autocorrelation coefficients). The trend and seasonal dummies do not seem to vary across clusters.

The final part of our web-based interactive tool is the forecast results displayed in Figs 5 and 6. We presented the forecasting performance on a one-week test set, using one OLS model in each cluster, and compared it with forecasts generated by ETS, a more complex method.

Error box and density plots in Fig 5 show the one-week-ahead forecast errors of three clusters using OLS and ETS models. Based on the error distribution of these two approaches, we can see that for the Taiwan COVID-19 dataset, OLS performs significantly better. We compute Pearson and concordance correlation coefficients between observed and forecasted values to evaluate the OLS performance and forecast precision on each cluster. Based on these coefficients, the forecasting result, computed by three OLS models, is precise. We also compared their performances using RMSE and MAE, and the results are the same as in plots. Lastly, we present two tables in Fig 6 that indicate the one-week-ahead forecast for all cities, townships, and districts in Taiwan using updated OLS and ETS models on combined training and test sets in each cluster.

## Conclusion

This research proposes an interactive web-based Shiny app for clustering and forecasting Taiwan COVID-19 confirmed infection cases. This tool is designed based on the MOB partitioning tree, cross-sectional attributes called domain-relevant attributes, and time series temporal patterns (trend, seasonality, and autocorrelation). Our tool helps users analyze Taiwan COVID-19 data via changing factors, including MOB depth, model complexity parameter (AIC or BIC), and domain-relevant attributes.

One advantage of our tool is grouping the series into interpretable clusters in which we can label a certain cluster by its corresponding domain-relevant attributes. This MOB-based clustering approach results in a single parametric OLS model in each cluster used to forecast all series in that cluster. Clustering series into groups with similar temporal patterns led us to enough accurate forecasts of Taiwan COVID-19 confirmed cases. This OLS forecasting approach has low computational complexity in forecasting these cases.

Our clustering results determine the different spread patterns of confirmed infection cases in the *least populated* in different regions and *most populated* areas. For example, the number of confirmed cases in populated areas is higher than in other places. Also, the COVID-19 time series shows different seasonality patterns on certain days of the week, higher on Sundays and lower on Mondays and Tuesdays.

Another advantage of our tool is its usefulness in handling the existence of missing values (missing completely at random (MCAR) or missing at random (MAR) variables)—displayed in gray in heatmaps. In addition, users can have the most updated results of the COVID-19 transmission in Taiwan by simply updating the dataset in the tool. Although this tool is specifically designed for Taiwan COVID-19 confirmed cases, it can be easily applied to other regions and/or countries with few changes and updates.

The OLS and ETS forecast results show an increase in infected cases in different cities. Note that these results are before vaccine rollout, and we need to adjust the model to consider the vaccination effect on the forecasting results. In addition, the concordance of our forecast is not studied in this work, and we expect that the forecast has a no-more-than moderate concordance. It is a future task to improve the concordance of our forecast.

## Supporting information

S1 File (PDF)
