# Supplementary material for: Interactive tool for clustering and forecasting patterns of Taiwan COVID-19 spread
Source: PLoS One. 2022 Jun 30;17(6):e0265477. doi: 10.1371/journal.pone.0265477 (PMC9246234; doi:10.1371/journal.pone.0265477)
Supplement: S1 File — (PDF) [file pone.0265477.s001.pdf]

The raw data was collected from Taiwan Centers for Disease Control (CDC) and it is available at www.cdc.gov.tw/En. The tool and dataset are publicly available at github.com/mahsaashouri/Taiwan-COVID-19-Interactive-tool. The population information mentioned in Table 1 is obtained from en.wikipedia.org/wiki/List_of_townships/cities_and_districts_in_Taiwan. We also created a binder interface [1] for our tool, which efficiently rinning the code in the browser.

Reference

1. Ragan-Kelley B, Willing C. Binder 2.0-Reproducible, interactive, sharable environments for science at scale. In: Proceedings of the 17th Python in Science Conference (F. Akici, D. Lippa, D. Niederhut, and M. Pacer, eds.); 2018. p. 113–120.
